# Testing models of speciation from genome sequences: divergence and asymmetric admixture in Island South-East Asian *Sus* species during the Plio-Pleistocene climatic fluctuations

**DOI:** 10.1111/mec.12958

**Published:** 2014-11-05

**Authors:** Laurent A F Frantz, Ole Madsen, Hendrik-Jan Megens, Martien A M Groenen, Konrad Lohse

**Affiliations:** * Animal Breeding and Genomics Group, Wageningen UniversityDe Elst 1, Wageningen, WD, 6708, The Netherlands; 2 Institute of Evolutionary Biology, University of EdinburghEdinburgh, EH93FL, UK

**Keywords:** conservation genetics, genomics, hybridization, phylogeography, population genetics, speciation

## Abstract

In many temperate regions, ice ages promoted range contractions into refugia resulting in divergence (and potentially speciation), while warmer periods led to range expansions and hybridization. However, the impact these climatic oscillations had in many parts of the tropics remains elusive. Here, we investigate this issue using genome sequences of three pig (*Sus*) species, two of which are found on islands of the Sunda-shelf shallow seas in Island South-East Asia (ISEA). A previous study revealed signatures of interspecific admixture between these *Sus* species (*Genome biology*,**14**, 2013, R107). However, the timing, directionality and extent of this admixture remain unknown. Here, we use a likelihood-based model comparison to more finely resolve this admixture history and test whether it was mediated by humans or occurred naturally. Our analyses suggest that interspecific admixture between Sunda-shelf species was most likely asymmetric and occurred long before the arrival of humans in the region. More precisely, we show that these species diverged during the late Pliocene but around 23% of their genomes have been affected by admixture during the later Pleistocene climatic transition. In addition, we show that our method provides a significant improvement over D-statistics which are uninformative about the direction of admixture.

## Introduction

Over the last four million years, the Earth has undergone frequent climatic oscillations including many ice ages (Zachos. 2001; Miller. 2005). Genetic studies have revealed that these large scale climatic fluctuations played a critical role in the evolutionary history of contemporary species (Hewitt 2000, 2004). Recent studies making use of the increased power afforded by genome-scale data have allowed to test increasingly finer hypotheses regarding the existence and the timing of post-divergence gene flow (i.e. Lawniczak. 2010; Rohland. 2010; Cahill. 2013; Hearn. 2014).

The impact that quaternary climatic fluctuations had on speciation is highly dependent on the taxa and the geographic range (Stewart. 2010). In many temperate regions, range contractions into refugia during glacial periods likely promoted divergence (and speciation), while range expansions out of refugia during warm periods resulted in hybridization. However, we know a lot less about the Pleistocene history of less well-studied biodiversity hotspots in the tropics (Hewitt 2004, 2011).

**Figure 1:**
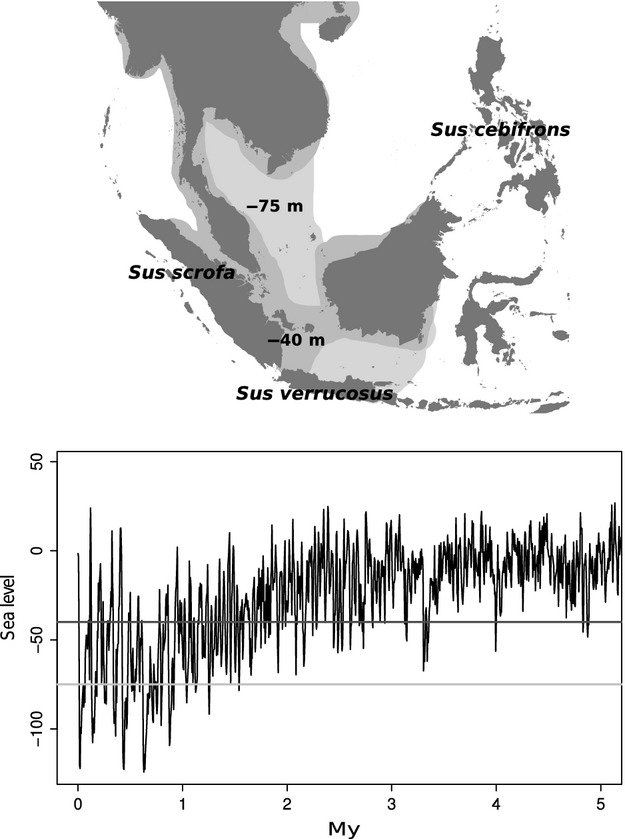
Map of Island South-East Asia (upper part) with sea level fluctuations (lower part) over the last 4My (adapted from Miller. 2005). Dark grey and light grey areas on the map represent the exposure of the Sunda-shelf at 40 m and 75 m below current sea level, respectively.

In this study, we investigate the history of divergence and admixture of three species of pigs (genus *Sus*) from Island South-East Asia (ISEA). The ISEA archipelago comprises thousands of islands on multiple tectonic plates (Hall 1998). While the islands of Borneo, Sumatra and Java and the Malay Peninsula form a large continental shelf known as the Sunda-shelf (Fig.1), other Island clusters such as the Philippines are on different plates. Islands on the same continental shelf are often separated by shallow seas and, given the large scale climatic fluctuations during the Pliocene and Pleistocene and the resulting sea level changes, were connected by land bridges on many occasions (Hall 1998; Voris 2000). In particular, the sharp climatic transition in the mid-Pleistocene (around 700KY) resulted in more frequent glacial cycles and hence exposure of the Sunda-Shelf (Elderfield. 2012). However the effect of this climatic transition on forest cover and the history of those species that depend on it, remains controversial (Gathorne-Hardy. 2002; Bird. 2005; Cannon. 2009; Wurster. 2010; Slik. 2011).

The aim of this study is to characterize the speciation history of pig species in the genus *Sus* in ISEA. We focus on three species: *Sus verrucosus* (Java warty pig; Java, Indonesia), *Sus cebifrons* (Visayan warty pig; Philippines) and *Sus scrofa* (Eurasian wildboar; mainland Eurasia, Sumatra and North Africa). Most species in the genus *Sus*, such as *S. verrucosus* and *S. cebifrons*, are endemic to a single or few islands of ISEA (Meijaard. 2011). In contrast, *S. scrofa* is a widely distributed species with a natural range extending to most of Eurasia, North Africa and part of ISEA (Sumatra; Meijaard. 2011). In addition, this species has been introduced by humans into multiple regions of the world such as North America, Australia and Java (Meijaard. 2011). A previous study showed that *S. verrucosus* from Java (Sunda-shelf) is more closely related to *S. cebifrons* (Visayan Warty pig) in the Philippines than to *S. scrofa* (Eurasian wild boar) on Sumatra (Sunda-shelf; Fig.1; Frantz. 2013). Moreover, this study showed that subsequent interspecific admixture likely took place on the Sunda-shelf, between *S. scrofa* and *S. verrucosus* after *S. cebifrons* diverged in the Philippines (Frantz. 2013). However, the timing, magnitude direction of admixture remains unknown. Firstly, it is unclear whether this interspecific admixture occurred naturally at all or, alternatively, whether it was the result of human-mediated translocation of pig species in ISEA (Groves 1984; Heinsohn 2003; Larson. 2005, 2007; Frantz. 2013) during the last 70 Ky (Mijares. 2010). This is crucial for conservation efforts such as *ex-situ* breeding programmes, particularly for the endangered Java Warty pig *S. verrucosus* (Semiadi. 2008) and the critically endangered Visayan warty pig *S. cebifrons* (Oliver 2008). Secondly, if admixture was natural, we would like to understand its temporal context. For example, both divergence and hybridization could be the result of the mid-Pleistocene sharp climatic transition. Alternatively, these species may have diverged much earlier during the late Pliocene or early Pleistocene, when connections between islands on the Sunda-shelf were less frequent (Frantz. 2013; Fig.1) and admixed again during the more frequent and intense glacial period of the latter Pleistocene.

In this study, we analysed three genomes of *Sus* from ISEA in a likelihood framework to i) determine whether interspecific admixture between *S. verrucosus* and *S. scrofa* is linked with recent human activities and ii) quantify the timing, extent and directionality of this admixture.

## Material and methods

### Data set

We used a genomic data set from three species of South-East Asian pigs that was previously analysed using phylogenetic methods and D-statistics (Frantz. 2013). The data set comprises a single unphased diploid genome sampled from a Eurasian wild boar *Sus scrofa* (Sumatran population; Fig.1) and the two island endemics *Sus verrucosus* (Java; Fig.1) and *Sus cebifrons* (Philippines; Fig.1). Triplet alignments were rooted using *Phacochoerus africanus* (common African warthog) as an outgroup. These genomes were sequenced at 10–20× depth of coverage and aligned to the *S. scrofa* reference genome (Ssc10.2; Bosse. 2012; Groenen. 2012; Frantz. 2013). The likelihood method we use fits explicit models of species divergence and admixture from multilocus data (see likelihood method) and requires short blocks of phased sequence (within which recombination can be ignored) of equal length (Lohse. 2011; Hearn. 2014; Lohse & Frantz 2014). We divided the reference genome of *S. scrofa* into 500 and 1000 bp blocks. To ensure enough coverage to call all heterozygous sites in each block and to remove possible copy number variants (Paudel. 2013), we filtered out, for each species, any block that had an average read depth of coverage lower than 7× or higher than twice the genomewide average (Frantz. 2013) using the pileup format in SAMtools v0.1.12 (Li. 2009). Clusters of two or more single nucleotide polymorphisms (SNPs) in a 10 bp window were filtered out as well as SNPs within 3 bp of an indel. We remo-ved blocks for which less than 90% of the sites were covered and excluded any site that had an effective coverage (Gronau. 2011) below 4. Lastly, we only selected blocks that passed the above filtering criteria in all four samples. We then randomly phased these diploid blocks as a previous study showed that maximum likelihood estimates (MLE; Lohse & Frantz 2014) are robust to phasing error provided blocks are short. Although the data were phased at random, the low heterozygosity – only 0.12% of sites were heterozygous in the *S. scrofa* individual from Sumatra, the most out-bred sample (Bosse. 2012; Frantz. 2013) – meant that the majority (67%) of 500 bp sequences alignments contained at most one heterozygous site per individual and so were immune to phasing error. Violations of the 4-gamete criterion within a block can arise either due to recombination or back mutation, both of which are not compatible with the assumption of the model (Lohse & Frantz 2014). We therefore excluded blocks containing more than one type of shared derived mutation (6.6% and 15.6% in the 500-bp and 1 kb data sets, respectively). After applying these filtering steps to the entire pig autosome, we were left with 232 373 and 190 692 of 500-bp and 1-kbp blocks, respectively.

### Models

**Figure 2:**
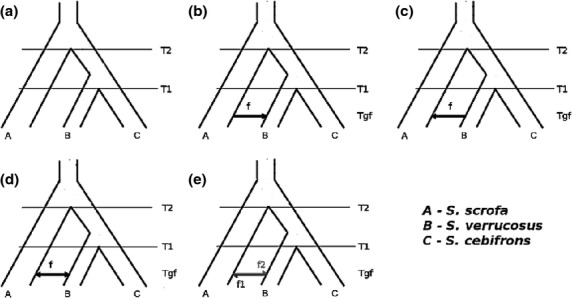
Schematic representation of the five models tested in this study: a) Strict divergence (DIV without admixture or divergence with) b) with admixture from *S. scrofa* to *S. verrucosus* (IUA_SS), c) admixture from *S. verrucosus* to *S. scrofa* (IUA_SV), d) symmetrical admixture (ISA) and e) bidirectional admixture (IBA).

We compared the fit of five nested models to test different scenarios for the evolutionary history of these species (Fig.2). All our models assume the order of species divergence inferred by Frantz. 2013 as (*S. scrofa*, (*S. cebifrons*,*S. verrucosus*)) and have at least three parameters, the species divergence time *T*_*1*_ (divergence of *S. verrucosus* and *S. cebifrons*), *T*_*2*_ (the species divergence of *S. scrofa* and *S. verrucosus*/*S. cebifrons*) and a single *N*_*e*_ parameter (constant effective population size). Based on D-statistics analysis (Green. 2010; Durand. 2011), we assumed that interspecific gene flow takes place between *S. verrucosus* and *S. scrofa* after the divergence of *S. cebifrons* (Frantz. 2013; Data S1, Supporting information). We first assessed the fit of the most complex/general history of instantaneous bidirectional admixture (IBA; Fig.2e), that is a scenario in which admixture between *S. scrofa* and *S. verrucosus* is assumed to happen in both directions. This model involves two admixture parameters (*f*_*1*_ and *f*_*2*_) and six parameters in total: *T*_*1*_, *T*_*2*_, *f*_*1*_, *f*_*2*_, *T*_*gf*_ (time of admixture) and *N*_*e*_. We then assessed the fit of different model simplifications: (i) a model of symmetrical admixture (ISA; Fig.2d, *f*_*1*_ = *f*_*2*_ = *f*) and (ii) models of instantaneous unidirectional admixture (IUA) Fig.2b and c) in which admixture goes only one way (either *S. scrofa* → *S. verrucosus* [IUA_SS] or *S. verrucosus → S. scrofa* [IUA_SV]). These are special cases of the IBA model in which we set either *f*_*1*_* *=* *0 or *f*_*2*_* *=* *0 (Fig.2) and so have five parameters. Lastly, we evaluated the support of a simple divergence model (DIV; Fig.2a) with no interspecific admixture, that is f_*1*_ = *f*_*2*_* *=* *0.

The assumption of equal population size for all these species may be unrealistic. To test whether adding additional demographic parameters improved model fit, we also evaluated the support of the IUA models with different population size. We tested additional models in which we allowed either *S. scrofa* or both *S. verrucosus* and the ancestral population of *S. verrucosus* and *S. cebifrons* to have a different *N*_*e*_ than the common ancestor of all three species (note that given the sampling scheme, we have no information about the *N*_*e*_ of *S. cebifrons)*. Because a model with two *N*_*e*_ parameters and two admixture fractions is nonidentifiable with minimal samples, we only assessed the influence of extra *N*_*e*_ parameters on the IUA models (IUA_SS and IUA_SV; Table S1, Supporting information).

### Likelihood analysis

Polymorphism information in a block of sequences from three species can be summarized as a vector of counts of mutations on different genealogical branches. For a polarized sample (know ancestral state) of three sequences, there are six possible mutations types: three private and three shared mutations. We will hereafter refer to this vector as the mutational configuration of a block. Lohse. (2011, eqn 1) have shown that the probability of an observed mutational configuration in a particular block can be expressed as a higher order derivative of the generating function of genealogical branch lengths. The generating function for triplet samples for the IUA models is described in Hearn. (2014) and Lohse & Frantz (2014). We give analogous results for the more general case of the IBA model in the supplementary information (Data S1, Supporting information). Assuming (initially) that blocks are unlinked (hence statistically independent observations), the logarithm of the likelihood (lnL) for a particular model is the sum of the lnL across blocks. We maximize the likelihood numerically using *Mathematica* v10. To correct for the effect of linkage when comparing models, we rescaled the difference in lnL between models as described in Lohse & Frantz (2014). We assumed that the effect of physical linkage between blocks separated by a distance of 100 kb can be ignored (Tortereau. 2012). Further details of the general method for computing likelihoods and 95% CI of parameters are given in Lohse. (2011) and Lohse & Frantz (2014). For each model, we computed *ΔlnL*, the difference in log likelihood to the best fitting model. We assessed statistical support between nested models with a likelihood ratio test and assumed that 2*ΔlnL follows a χ^*2*^ distribution with degree of freedom equal to the difference in the number of parameters of the two models (see Table1).

**Table 1:** Model description and difference in support compared to best fitting model for 500 bp and 1 kb blocks

Acronym (parameters)	Description	ΔlnL (500)	ΔlnL (1k)
DIV (3)	Strict divergence (no gene flow) with or without ancestral substructure (Fig.2a)	−49.3^*^^*^	−80.1^*^^*^
IUA_SS (5)	Divergence with gene flow from *S. scrofa* to *S. verrucosus* (Fig.2b)	−22.8^*^^*^	−26.8^*^^*^
IUA_SV (5)	Divergence with gene flow from *S. verrucosus* to *S. scrofa* (Fig.2c)	−1.7 N.S.	−1.6 N.S.
ISA (5)	Symmetrical admixture model with equal admixture fractions (Fig.2d)	−10.1^*^^*^	−9.24^*^^*^
IBA (6)	Bidirectional admixture model with independent admixture fraction (Fig.2e)	0	0

Significance was obtained using a likelihood ratio test (2^*^ΔlnL) and assuming a chi-square distribution (^*^^*^P < 0.001; ^*^ *P* < 0.01)

To compare our approach with the D-statistics (Green. 2010; Durand. 2011) and to obtain a rough assessment of goodness of fit, we computed the expected counts of ABBA and BABA sites and E[D] from the generating function under the different admixture models (by fixing parameters to their MLE estimated from the data; see Data S1, Supporting information).

To scale relative time estimates into absolute values, we assumed an average divergence time between the African warthog and the ingroup of 10.5 MY and a generation time of 5 years (Gongora. 2011; Groenen. 2012; Frantz. 2013).

## Results

### Model comparison

By definition, the IBA model (Fig.2e) provided a better fit than any of the simpler nested models (Table1). Setting admixture fractions to be equal (*f*_*1*_ = *f*_*2*_ = *f*; Fig.2d) significantly reduced the fit (ISA model; *Δ;lnL *= −10.1 and −9.24 for 500 and 1 kb, respectively; Table1). This difference in likelihood is highly significant assuming a χ^*2*^ distribution (*P* < 0.001; Table1). Likewise, a model in which *f*_*1*_* *=* *0 (IUA_SS; Fig.2c), that is a history with admixture only from *Sus scrofa* into *Sus verrucosus* also gave a significantly worse fit (*Δ;lnL *= −22.8 and −26.8 for 500 and 1 kb, respectively; Table1). In contrast, setting *f*_*2*_* *=* *0 (IUA_SV model; Fig.2b) only marginally reduced the fit (*Δ;lnL *= −1.77 [*P* > 0.05] and −1.68 [*P* > 0.05] for 500 and 1 kb, respectively; Table1). Thus, a model of unidirectional admixture from *S. verrucosus* into *S. scrofa* cannot be rejected.

Lastly, a strict divergence model, that is *f*_*1*_ = *f*_*2*_* *=* *0 (DIV model; Fig.2a) provided a significantly worse fit (*Δ;lnL *= −49.3 and −80.1 for 500 and 1 kb, respectively; Table1). These results demonstrate that this genomic data set contains a strong signal of interspecific admixture between *S. scrofa* and *S. verrucosus,* but surprisingly most of this admixture occurred from *S. verrucosus* into *S. scrofa,* so in the opposite direction than that assumed by previous studies (Frantz. 2013).

**Figure 3:**
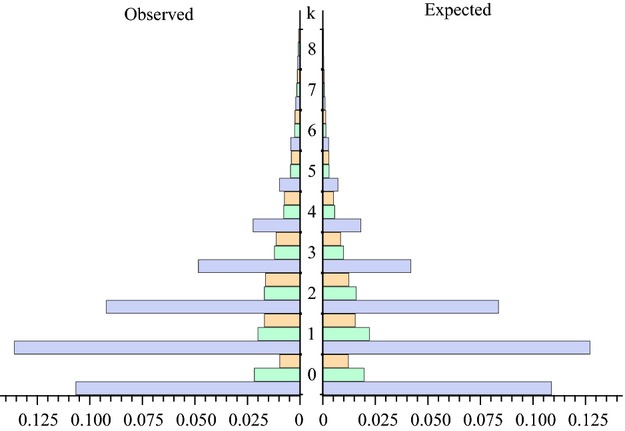
Expected (under IBA model with parameters fixed at their MLE) and observed mutational configuration. The *X*-axis represents the proportion of blocks with k mutations (Y axis) for different topologies. Blue bars = (*S. scrofa*, (*S. verrucosus*,*S. cebifrons*)), green bars = (*S. verrucosus*, (*S. cebifrons*,*S. scrofa*)) and orange = (*S. cebifrons*, (*S. verrucosus*,*S. scrofa*)).

Including additional *N*_*e*_ parameter for different populations (see Methods) did not significantly improve the fit (Table S1, Supporting information). To get a sense of how well different admixture histories explain the data, we computed the expected D statistic (E[D]) under each admixture scenario and compared it to the observed value. This also allowed us to assess the sensitivity of D to admixture in different directions. Constraining admixture to be from *S. scrofa* to *S. verrucosus* (the best fitting IUA_SV model) gives E[D] = 0.22, while limiting admixture to the opposite direction only (the IUA_SS model) gives E[D] = 0.12. The observed D of 0.175 (for 0.5 kb data) is in between and matches E[D] under the estimated IBA model E[D] = 0.16. Thus, the unidirectional models both fit the data worse than the bidirectional scenario (IBA). Comparing the number of mutations on external branches for each of the three possible topologies expected under the IBA model to the observed spectrum of mutation counts reveals a tight fit (Fig.3), suggesting that the IBA model explains most of the signal in the data.

### Parameter inference

**Figure 4:**
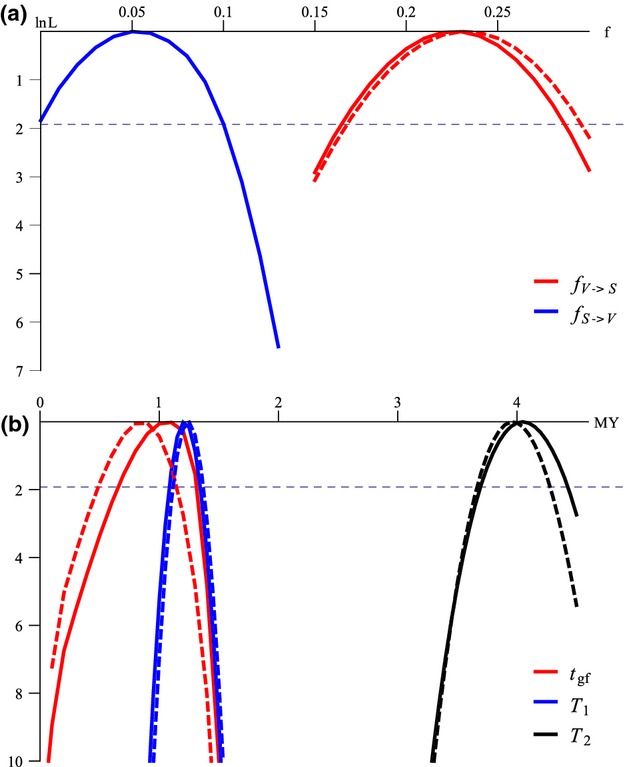
Marginal support (*Δ;lnL*) for a) admixture fraction (*f*) and b) divergence and admixture times. Solid and dashed lines represent *Δ;lnL* curves under the IUA_SV and IBA models, respectively. The black dashed horizontal lines delimit the 95% confidence interval. a) admixture fraction from *S. verrucosus* into *S. scrofa* and from *S. scrofa* into *S. verrucosus* are shown in red and blue represent respectively b) *T*_*2*_, *T*_*1*_ and *T*_*gf*_ are shown in black, blue and red respectively.

Maximum-likelihood estimates for each parameter (Fig.4) were obtained under the best fitting models (IBA and IUA_SV). Our first goal was to determine whether the admixture between *S. scrofa* and *S. verrucosus* could have been mediated by humans. The marginal curves for the time of admixture, under both IBA and IUA_SV models, show very little support for values of *T*_*gf*_ below 70 Ky (*Δ;lnL* < −6; Fig.4b), the time of the earliest human arrival in the region (Mijares. 2010), which strongly suggest that humans did not play a role in this admixture.

Our second goal was to put the initial divergence between these species in the context of the climate history during the Pleistocene. Our point estimate for *T*_*2*_, *that is* the deeper speciation event in this study (*S. scrofa* and *S. verrucosus*/*Sus cebifrons* split, Fig.2) is approximately 4My (Fig.4b) for both block sizes, with lower 95% CI much older than >2.5My (Plio-Pleistocene transition; Fig.4b). We estimated the split between the Javan warty pig (*S. verrucosus*) and the Visayan warty pig (*S. cebifrons*) (*T*_*1*_) to be between 1.3 and 1.1My (Fig.4b) These divergence time estimates agree well with previous analyses based on the same molecular clock (Frantz. 2013).

## Discussion

In this study, we show that the extent and the directionality of interspecific admixture between Sunda-shelf *Sus* species (Fig.1) is more complex than previously assumed (Frantz. 2013). First, our likelihood approach allows us to rule out any major influence of humans in this admixture event. Second, our analysis suggests that ISEA *Sus* species diverged during warmer periods of the late Pliocene and hybridized during the more frequent glacial periods of the mid-Pleistocene. Finally, and perhaps surprisingly, our analyses suggest that admixture occurred mainly from *Sus verrucosus* into *Sus scrofa,* so in the opposite direction than that assumed previously.

### Fine scale model testing using a likelihood approach

Models involving interspecific admixture between *Sus* species on the Sunda-shelf (Fig.1) fitted our genomic data set significantly better than a simple divergence model without gene flow (Table1). We estimated a 23% admixture fraction from the Java Warty pig (*S. verrucosus*) into the *S. scrofa* population on Sumatra (Fig.4). This admixture can explain the large discrepancies found between nuclear and mtDNA phylogenetic analyses that found that *S. verrucosus* and Sumatran *S. scrofa* share very similar mtDNA haplotypes and form a monophyletic clade with short external branches (Larson. 2005, 2007; Frantz. 2013). Moreover, it seems that replacement of mtDNA in either *S. verrucosus* or *S. scrofa* from Sumatra was complete as no divergent mtDNA haplotypes have been found by any previous studies (Larson. 2005, 2007). This suggests that interspecific admixture can lead to complete (or near-complete) mtDNA replacement even with an admixture fraction of ∽ 23% and illustrates that phylogenetic and phylogeographic studies that rely solely on mtDNA can be highly misleading.

Our results also show that previous analyses of these genomes (Frantz. 2013), using D-statistics (Green. 2010; Durand. 2011), only incompletely resolved this interspecific admixture. This is because the D-statistics provide no direct information about the direction of admixture (Green. 2010). Frantz. 2013 had assumed a history of unidirectional admixture from *S. scrofa* into *S. verrucosus* to be able to compute an upper bound for the admixture fraction under this model from ABBA/BABA counts (see Additional File 6 in Frantz. 2013). In contrast, the joint distribution of branch lengths, used in our likelihood approach, contains additional information about the direction of admixture and our analysis reveals that *S. verrucosus* is largely the source of admixture rather than the recipient. While we can show that the IBA model fitted slightly better than the IUA_SV model, the difference was not significant (Table1). In other words, although our estimate for *f* (IBA model) from *S. scrofa* to *S. verrucosus* was very similar to the fraction estimated by Frantz. 2013 (4% vs. 5% in this study), our 95% CI for this parameter also include 0 (Fig.4a). Therefore, while our results unequivocally support that *S. verrucosus* was the most important source of interspecific admixture, we cannot rule out that this species was also a recipient. Together our analyses show that it is important to interpret admixture fractions computed based on D-statistics with caution when the direction of the admixture is unknown.

### Natural interspecific admixture on the Sunda-shelf

Our analysis showed that most of the interspecific admixture between the Sunda-shelf species took place before humans arrived in the region and so anthropogenic disturbances are unlikely to explain this phenomenon. However, while useful, our models are necessarily oversimplistic. For example, we assumed that admixture was a single, instantaneous event. However, the Sunda-shelf was exposed during multiple glacial cycles in the mid-Pleistocene (Voris 2000), which in turn could have lead to many admixture events. Therefore, although we ruled out humans as the cause for most of this admixture, this does not exclude the possibility that a small amount of admixture occurred more recently as a result of anthropogenic disturbances. This is especially true given that previous studies have found that humans most likely translocated *Sus* species in the region (Groves 1984; Heinsohn 2003; Larson. 2005, 2007; Frantz. 2013). Thus, the genomic signature left by human-mediated translocation of species may be confounded with the signal of large-scale, naturally occurring admixture.

We also evaluated the support of a model of symmetrical admixture (ISA; Table1). Our model comparison clearly demonstrates that this scenario provides a significantly poorer fit than the IBA model. This is unsurprising given the large difference in admixture fraction under the IBA model (5% vs. 23%). This asymmetry could arise in at least two ways: First, a low effective population size (*N*_*e*_) of Sumatran *S. scrofa* – perhaps as a result of a founder event when this species colonized Sumatra from the mainland at the time of admixture could explain this observation. However, the origin of *S. scrofa* (on the mainland or on ISEA) remains controversial due to the difficulty of inferring demographic events that took place more than 2My ago (Frantz. 2013). Alternatively, this discrepancy in the admixture fraction could be the result of greater mate discrimination against hybrids by *S. verrucosus*. This interpretation is difficult to assess given the very sparse ecological and behavioural data available for the Javanese warty pig *S. verrucosus* (Blouch 1993). However, such information is available for *S. scrofa*, an invasive generalist that can easily colonize new environments (Barrios-Garcia & Ballari 2012). *Sus scrofa* can be found natively all over Eurasia, in Sumatra and parts of North Africa. Moreover, feral *S. scrofa* have been able to colonize new ecosystems in Australia, Hawaii, Java, North America and many other parts of the globe in recent years. Thus, given the generalist behaviour of this species, its wide range and its ability to colonize new environments and the relatively narrow range of *S. verrucosus* (restricted to a few areas on Java) mate discrimination against hybrids by the latter would appear more probable. Such an asymmetry in sexual selection against hybrids has been suggested in mice (i.e. Latour. 2014). Further research on the ecology and the behaviour of the Java warty pig is needed to better interpret these results, especially given its endangered status. More efficient mate discrimination against hybrids by *S. verrucosus* would have important consequences for on-going conservation effort. Indeed, one of the major threats to *S. verrucosus*, listed by the IUCN Wild Pig Specialist Group (Semiadi. 2008), is the hybridization with the potentially recently introduced *S. scrofa* on Java. However, hybrids are difficult to identify in the wild and the extent of this threat remains unknown (Semiadi. 2008). Disentangling these two hypotheses (hybrid recruitment vs. founder effect) would provide crucial information for the conservation of *S. verrucosus*.

Adding parameters to model population size difference between these species did not improve the fit of our models (Table S1, Supporting information). This does of course not imply that these species have the same effective population size, but rather demonstrates that there is little information in the blockwise data to fit more realistic histories (Fig.3). In contrast, the information contained in linkage across longer stretches of the genome suggests that these species have experienced substantial changes in *N*_*e*_. For example, both *S. verrucosus* and *S. cebifrons* carry long runs of homozygosity and have a low current *N*_*e*_ that was attributed to very recent bottlenecks possibly due to anthropogenic disturbances (Bosse. 2012; Frantz. 2013). In addition, all three species showed demographic signals consistent with long-term bottlenecks during the Pleistocene (Frantz. 2013). Lastly, as discussed above, in the IBA model, *f* and *N*_*e*_ are almost entirely confounded (Lohse. 2011), thus it is not surprising that there is no additional power to estimate variation in the latter.

### The impact of Plio-Pleistocene glaciations on the evolutionary history of Sus

Our analyses show that the divergence between Sunda-shelf *Sus* species took place during the Pliocene around 4My ago (Fig.4b). Moreover, we found that the divergence between *S. verrucosus* and *Sus cebrifrons* occurred over 1.2My ago, during the early Pleistocene before the sharp climatic transition ∽700Ka (Fig.4b). This suggests that the milder climatic fluctuations of the Pliocene (Fig.1) allowed for dispersal between ‘islands’ during short glacial period and subsequent isolation during long warm periods (due to high sea level), while the longer and more frequent ice ages of the late Pleistocene which resulted in longer exposure of the Sunda-shelf (due to low sea level) led to a partial merging of gene pools. Thus, the Plio-Pleistocene climatic fluctuations may have had the reverse effect in ISEA compared to more temperate regions such as Europe, in which glacial maximas induce divergence (assemblage of refugia) and interglacial periods induce range expansions and hybridization (*i.e*. Hewitt 2000, 2004, 2011; Schmitt 2007). However, our large confidence intervals around time parameters (divergence and admixture) as well as our model assumptions (single instantaneous admixture) do not provide the necessary resolution to correlate these events with individual glacial cycles during the Plio-Pleistocene era (Zachos. 2001; Elderfield. 2012). Further studies using larger data sets, increasingly sophisticated methods and combining historical inferences from multiple species will shed light on the mechanisms that generated and erased biodiversity in this mega biodiverse region of the world (Myers. 2000).

## Data accessibility

BAM files: EBI Sequence Read Archive ERP001813.

Mathematica notebook and input files (mutational configurations for 500-bp and 1-kbp blocks): Dryad doi:10.5061/dryad.1q0h6.

## Supporting Information

**Table S1.** Support for model with extra *N*_*e*_ parameters.

**Data S1.** Supporting *Mathematica* notebook.
