# Supplementary material for: Testing models of speciation from genome sequences: divergence and asymmetric admixture in Island South-East Asian Sus species during the Plio-Pleistocene climatic fluctuations
Source: Mol Ecol. 2014 Nov 5;23(22):5566–74. doi: 10.1111/mec.12958 (PMC4245187; doi:10.1111/mec.12958)
Supplement: Supplementary file 1 — Table S1. Support for model with extra Ne parameters. [file mec0023-5566-sd1.doc]

**Table S1:** Full model descriptions and support for 500bp and 1kb blocks.

| Acronym | Description | *ΔlnL* (500bp) | *ΔlnL* (1kbp) |
| --- | --- | --- | --- |
| DIV | Strict divergence (no gene-flow) with or without ancestral substructure (Figure 2a) | 49.3 | 80.1 |
| IUA_SS | Divergence with gene-flow from *S. scrofa* to *S. verrucosus* (Figure 2b) | 22.8 | 26.8 |
| IUA_SSα | Same as above but with an extra Ne parameters for *S. scrofa* (Figure 2b) | 21.6 | 20 |
| IUA_SSβ | Same as above but with two extra Ne parameters for S. verrucosus and S. verrucosus/S. cebifrons ancestral population (Figure 2b) | 20.7 | 19.7 |
| IUA_SV | Divergence with gene-flow from *S. verrucosus* to *S. scrofa* (Figure 2c) | 1.77 | 1.68 |
| IUA_SVα | Same as above but with an extra Ne parameters for *S. scrofa* (Figure 2c) | 1.77 | 3.09 |
| IUA_SVβ | Same as above but with two extra Ne parameters for S. verrucosus and S. verrucosus/S. cebifrons ancestral population (Figure 2c) | 1.77 | 3.09 |
| ISA | Symmetrical admixture model with equal admixture fractions. (Figure 2d) | 10.1 | 9.24 |
| IBA | Bi-directional admixture model with independent admixture fraction (Figure 2e) | **0** | **0** |
