# Supplementary material for: Testing models of speciation from genome sequences: divergence and asymmetric admixture in Island South-East Asian Sus species during the Plio-Pleistocene climatic fluctuations
Source: Mol Ecol. 2014 Nov 5;23(22):5566–74. doi: 10.1111/mec.12958 (PMC4245187; doi:10.1111/mec.12958)
Supplement: Supplementary file 2 — Data S1. Supporting Mathematica notebook. [file mec0023-5566-sd2.pdf]

Testing models of speciation from genome sequences: divergence and asymmetric admixture in Island Southeast Asian *Sus* species during the Plio-Pleistocene climatic fluctuations - Supporting notebook

Deriving the generating function of genealogical branch lengths for the IBA model

The generating function (GF) for a model of bi-directional admixture is a simple extension of the unidirectional case given in Lohse & Frantz (2014). We use the general recursion for the generating function (Lohse et al. 2011, eq. 3) of genealogical branches to trace the history of a triplet sample (a,b,c) backwards in time and initially consider a model where divergence and admixture events i are exponentially distributed random variables with rates Λ_i (rather than discrete events). The first event in the history of the sample is admixture between popultaion A and B which happens at time Λ_{gf}. We denote the configuration at the time of sampling *(a/b/c) (where the asterisk indicates that the admixture event is still pending), the admixture fraction from population A to B (forwards in time) f_a and admixture from population B to A f_b. There are four possible sampling configurations after the admixture event:

$f_a(1-f_b)$	(a,b/Ø/c)
$f_b(1-f_a)$	(Ø/a,b/c)
$f_b f_a$	(b/a/c)
$(1-f_b)(1-f_a)$	(a/b/c)

We use Ø top denote an empty population. So the GF after for the first (going backwards) event in the history of the sample is:

$$\psi[*(a/b/c)] == \frac{\Lambda[\{GF\}]}{\Lambda[\{GF\}] + \omega[\{a\}] + \omega[\{b\}] + \omega[\{c\}]} (f_a (1-f_b)\, \psi[(a,\, b/Ø/c)] + f_b (1-f_a)\, \psi[(Ø/a,\, b/c)] + f_a f_b\, \psi[(b/a/c)] + (1-f_b)(1-f_a)\, \psi[(a/b/c)])$$

The remaining sampling configurations and corresponding GF equations are identical to those under the models considered by Hearn et al. (2014). To allow for different effective population sizes in populations A and B, we scale the rate of pairwise coalescence in these populations by α and β respectively. The full set of GF equations for the IBA model is:

```
In[1]:= neanderDurandbothαβ = {ψ[{{a}}, {{b}}, {{c}}, gf] == Λ[{GF}] / (Λ[{GF}] + ω[{a}] + ω[{b}] + ω[{c}])
    (fa (1 - fb) ψ[{}, {{a}, {b}}, {{c}}] + fb (1 - fa) ψ[{{a}, {b}}, {}, {{c}}] +
```

$$f_a f_b \psi[\{\{b\}\},\{\{a\}\},\{\{c\}\}] + (1-f_b)(1-f_a)\psi[\{\{a\}\},\{\{b\}\},\{\{c\}\}]),$$

$$\psi[\{\},\{\{a\},\{b\}\},\{\{c\}\}] == \frac{1}{\Lambda[\{1\}]+\beta+\omega[\{a\}]+\omega[\{b\}]+\omega[\{c\}]}(\Lambda[\{1\}]\psi[\{\{a\},\{b\},\{c\}\},\Lambda[\{2\}]]+\beta\psi[\{\},\{\{a,b\}\},\{\{c\}\}]),$$

$$\psi[\{\{a\},\{b\}\},\{\},\{\{c\}\}] == \frac{1}{\Lambda[\{1\}]+\alpha+\omega[\{a\}]+\omega[\{b\}]+\omega[\{c\}]}(\Lambda[\{1\}]\psi[\{\{a\},\{b\}\},\{\{c\}\},\alpha]+\alpha\psi[\{\{a,b\}\},\{\},\{\{c\}\}]),$$

$$\psi[\{\{a\},\{b\}\},\{\{c\}\},\alpha] == \frac{1}{\Lambda[\{2\}]+\alpha+\omega[\{a\}]+\omega[\{b\}]+\omega[\{c\}]}(\Lambda[\{2\}]\psi[\{\{a\},\{b\},\{c\}\}]+\alpha\psi[\{\{a,b\}\},\{\{c\}\}]),$$

$$\psi[\{\{a,b\}\},\{\},\{\{c\}\}] == \frac{\Lambda[\{1\}]}{\Lambda[\{1\}]+\omega[\{c\}]+\omega[\{a,b\}]}\psi[\{\{a,b\}\},\{\{c\}\}],$$

$$\psi[\{\{b\}\},\{\{a\}\},\{\{c\}\}] == \frac{\Lambda[\{1\}]}{\Lambda[\{1\}]+\omega[\{a\}]+\omega[\{b\}]+\omega[\{c\}]}\psi[\{\{b\}\},\{\{a\},\{c\}\}],$$

$$\psi[\{\{b\}\},\{\{a\},\{c\}\}] == \frac{1}{\Lambda[\{2\}]+\beta+\omega[\{a\}]+\omega[\{b\}]+\omega[\{c\}]}(\Lambda[\{2\}]\psi[\{\{a\},\{b\},\{c\}\}]+\beta\psi[\{\{b\}\},\{\{a,c\}\}]),$$

$$\psi[\{\{b\}\},\{\{a,c\}\}] == \frac{\Lambda[\{2\}]}{\Lambda[\{2\}]+\omega[\{b\}]+\omega[\{a,c\}]}\psi[\{\{b\},\{a,c\}\}],$$

$$\psi[\{\},\{\{a,b\}\},\{\{c\}\}] == \frac{\Lambda[\{1\}]}{\Lambda[\{1\}]+\omega[\{a,b\}]+\omega[\{c\}]}\psi[\{\{c\},\{a,b\}\}],$$

$$\psi[\{\{a\}\},\{\{b\}\},\{\{c\}\}] == \frac{\Lambda[\{1\}]}{\Lambda[\{1\}]+\omega[\{a\}]+\omega[\{b\}]+\omega[\{c\}]}(\psi[\{\{a\}\},\{\{b\},\{c\}\}]),$$

$$\psi[\{\{a\},\{b\}\},\{\{c\}\},\beta] == \frac{1}{\beta+\Lambda[\{2\}]+\omega[\{a\}]+\omega[\{b\}]+\omega[\{c\}]}(\Lambda[\{2\}]\psi[\{\{a\},\{b\},\{c\}\}]+\beta\psi[\{\{a,b\}\},\{\{c\}\}]),$$

$$\psi[\{\{a,b\}\},\{\{c\}\}] == \frac{\Lambda[\{2\}]}{\Lambda[\{2\}]+\omega[\{c\}]+\omega[\{a,b\}]}\psi[\{\{c\},\{a,b\}\}],$$

$$\psi[\{\{a\}\},\{\{b\},\{c\}\}] == \frac{1}{\beta+\Lambda[\{2\}]+\omega[\{a\}]+\omega[\{b\}]+\omega[\{c\}]}(\Lambda[\{2\}]\psi[\{\{a\},\{b\},\{c\}\}]+\beta\psi[\{\{a\}\},\{\{b,c\}\}]),$$

$$\psi[\{\{a\},\{b\},\{c\}\},\Lambda[\{2\}]] == \frac{1}{3\beta+\Lambda[\{2\}]+\omega[\{a\}]+\omega[\{b\}]+\omega[\{c\}]}(\beta\psi[\{\{c\},\{a,b\}\}]+\beta\psi[\{\{b\},\{a,c\}\}]+\beta\psi[\{\{a\},\{b,c\}\}]+\Lambda[\{2\}]\psi[\{\{a\},\{b\},\{c\}\}]),$$

$$\psi[\{\{a\},\{b\},\{c\}\}] == \frac{1}{3+\omega[\{a\}]+\omega[\{b\}]+\omega[\{c\}]}(\psi[\{\{c\},\{a,b\}\}]+\psi[\{\{b\},\{a,c\}\}]+\psi[\{\{a\},\{b,c\}\}]),$$

$$\psi[\{\{a\}\},\{\{b,c\}\}] == \frac{1}{\Lambda[\{2\}]+\omega[\{a\}]+\omega[\{b,c\}]}(\Lambda[\{2\}]\psi[\{\{a\},\{b,c\}\}]),$$

$$\psi[\{\{c\},\{a,b\}\},\Lambda[\{2\}]] == \frac{1}{\beta+\Lambda[\{2\}]+\omega[\{c\}]+\omega[\{a,b\}]}(\Lambda[\{2\}]\psi[\{\{c\},\{a,b\}\}]+\beta),$$

$$\psi[\{\{b\},\{a,c\}\},\Lambda[\{2\}]] == \frac{1}{\beta+\Lambda[\{2\}]+\omega[\{b\}]+\omega[\{a,c\}]}(\Lambda[\{2\}]\psi[\{\{b\},\{a,c\}\}]+\beta),$$

```
ψ[{{a}, {b, c}}, Λ[{2}]] == 1/(β + Λ[{2}] + ω[{a}] + ω[{b, c}]) (Λ[{2}] ψ[{{a}, {b, c}}] + β),
ψ[{{c}, {a, b}}] == 1/(1 + ω[{c}] + ω[{a, b}]),
ψ[{{b}, {a, c}}] == 1/(1 + ω[{b}] + ω[{a, c}]),
ψ[{{a}, {b, c}}] == 1/(1 + ω[{a}] + ω[{b, c}])} /. f_a → fa /. f_b → fb;
```

Conditioning on topology

We can partition the GF into contribution from the three possible topologies. topeq sets GF equations that contain ω variables that are incompatible with a given topology to 0. We also set $\beta = \alpha =1$ (i.e. assuming that all populations have the same effective size) to simplify:

```
In[2]:= topeq[l_List, t_List] :=
   Module[{eqvars}, eqvars = Table[Cases[Variables[l[[i, 2]]], ω[_]], {i, 1, Length[l]}];
    Table[If[Length[Intersection[t, eqvars[[i]]]] > 0, ReplacePart[l[[i]], 2 → 0], l[[i]]],
     {i, 1, Length[eqvars]}]];
```

```
In[3]:= neanderDurandbothαβbc = topeq[neanderDurandbothαβ /. α → 1 /. β → 1, {ω[{a, c}], ω[{a, b}]}];
neanderDurandbothαβab = topeq[neanderDurandbothαβ /. α → 1 /. β → 1, {ω[{a, c}], ω[{b, c}]}];
neanderDurandbothαβac = topeq[neanderDurandbothαβ /. α → 1 /. β → 1, {ω[{a, b}], ω[{b, c}]}];
```

Solving the set of GF equations for each topology:

```
In[5]:= neanderDurandbothαβbcsol =
   (Solve[neanderDurandbothαβbc, First /@ neanderDurandbothαβbc][[1, 1, 2]]) // Simplify;
neanderDurandbothαβabsol =
   (Solve[neanderDurandbothαβab, First /@ neanderDurandbothαβab][[1, 1, 2]]) // Simplify;
neanderDurandbothαβacsol =
   (Solve[neanderDurandbothαβac, First /@ neanderDurandbothαβac][[1, 1, 2]]) // Simplify;
```

We can take the InverseLaplaceTransform wrt to each of the three Λ variables to find the GF of the IBA model with discrete times:

```
In[8]:= neanderDurandbothGFbcαβ = InverseLaplaceTransform[Λ[{2}]^-1 InverseLaplaceTransform[Λ[{1}]^-1
       InverseLaplaceTransform[Λ[{GF}]^-1 neanderDurandbothαβbcsol, Λ[{GF}], T[{GF}]],
     Λ[{1}], T[{1}]], Λ[{2}], T[{2}]] // Simplify;
```

```
In[9]:= neanderDurandbothGFabαβ = InverseLaplaceTransform[Λ[{2}]^-1 InverseLaplaceTransform[Λ[{1}]^-1
       InverseLaplaceTransform[Λ[{GF}]^-1 neanderDurandbothαβabsol, Λ[{GF}], T[{GF}]],
     Λ[{1}], T[{1}]], Λ[{2}], T[{2}]] // Simplify;
```

```
In[10]:= neanderDurandbothGFacαβ = InverseLaplaceTransform[Λ[{2}]^-1 InverseLaplaceTransform[
     Λ[{1}]^-1 InverseLaplaceTransform[Λ[{GF}]^-1 neanderDurandbothαβacsol,
      Λ[{GF}], T[{GF}]], Λ[{1}], T[{1}]], Λ[{2}], T[{2}]] // Simplify;
```

The models with unidirectional admixture (IUA) and symmetric admixture (ISA) are nested within the IBA model, i.e. are special cases. I saves time to define the

Topological probabilities

Setting all remaining dummy variables of the GF conditional on topology to zero gives topological probabilities. An alterna - tive derivation of these probabilities can be made using discrete -time transition matrices (analogous to Slatkin and Pollack 2008). Note that for simplicity, in the GF expressions, the times of all discrete events are scaled from the previous event (rather than the present). So topological probabilities only depend on the time interval between the admixture and the population divergence:

```
In[11]:= neandertopoDurandbothαβ =
           {neanderDurandbothGFbcαβ, neanderDurandbothGFabαβ, neanderDurandbothGFacαβ} /.
             {ω[_] → 0} // FullSimplify
```

Out[11]=

$$\left\{\frac{1}{3} e^{-T[\{1\}]-T[\{2\}]} \left(3 e^{T[\{1\}]+T[\{2\}]} (-1+fa)(-1+fb) - e^{T[\{2\}]} fa (-1+fb) + fb - fa\, fb + e^{T[\{1\}]} (-2+2 fa+2 fb - fa\, fb)\right),\right.$$
$$\frac{1}{3} e^{-T[\{1\}]-T[\{2\}]} \left(2 e^{T[\{2\}]} fa (-1+fb) + 2(-1+fa) fb + 3 e^{T[\{1\}]+T[\{2\}]} (fa+fb-2 fa\, fb) + e^{T[\{1\}]} (1-fb+fa(-1+2 fb))\right),$$
$$\left.\frac{1}{3} e^{-T[\{1\}]-T[\{2\}]} \left(-e^{T[\{2\}]} fa (-1+fb) + fb - fa\, fb + 3 e^{T[\{1\}]+T[\{2\}]} fa\, fb - e^{T[\{1\}]} (-1+fa+fb+fa\, fb)\right)\right\}$$

▫ *Checks*

The topological probabilities must sum to one.

```
Total[neandertopoDurandbothαβ] // Simplify
```

1

Without admixture we get the well known topological probabilities (Takahata 1991) and symmetry between ab and ac topologies:

```
neandertopoDurandbothαβ /. fb → 0 /. fa → 0 // Simplify
```

$$\left\{\frac{1}{3}\left(3 + 2 e^{-T[\{2\}]} (-1+f) - 3 f + e^{-T[\{1\}]} f\right),\right.$$
$$\left.\frac{1}{3}\left(-e^{-T[\{2\}]} (-1+f) + 3 f - 2 e^{-T[\{1\}]} f\right),\ \frac{1}{3} e^{-T[\{1\}]-T[\{2\}]}\left(-e^{T[\{1\}]} (-1+f) + e^{T[\{2\}]} f\right)\right\}$$

Data analysis

▫ *Loading the data*

Sampling loci of length 500 with 1 kb distance between each pair gives 124, 474 loci. 'multiplesites', (0,0,0) (0,0,1), (0,1,0), (0,1,1), (1,0,0), (1,0,1), (1,1,0), (1,1,1) We are excluding loci with complex heterozygous sites...

```
In[51]:= rawpig = ReadList[
            "/home/konrad/Finished_Manuscripts/PigPhylogeography/Pig_data/500b_flt_counts.txt"][[
           1]];
         rawpig1kb = ReadList[
            "/home/konrad/Finished_Manuscripts/PigPhylogeography/Pig_data/1kb_flt_counts.txt"][[
           1]];
```

Need to re-order these (because the sample order is hard-coded):

```
In[53]:= rawpig11 = ({#1[[6]], #1[[3]], #1[[7]], #1[[4]], #1[[8]], #1[[5]]} &) /@ rawpig;
         rawpig111kb = {#[[6]], #[[3]], #[[7]], #[[4]], #[[8]], #[[5]]} & /@ rawpig1kb;
```

```
In[55]:= {rawpig11 // Length, rawpig111kb // Length}
```

```
Out[55]= {248 948, 226 080}
```

Summarizing the counts of mutational configurations:

```
In[59]:= pigfin1 = configcount3stt[rawpig11, 3];
         pigfin11kb = configcount3stt[rawpig111kb, 3];
```

The total number of blocks (0.5kb and 1 kb data)

```
Total[Flatten[#]] & /@ {pigfin1, pigfin11kb};
```

The proportion of blocks filtered out due to 4-gamete violations:

```
({rawpig11 // Length, rawpig111kb // Length} -
    (Total[Flatten[#]] & /@ {pigfin1, pigfin11kb})) /
  ({rawpig11 // Length, rawpig111kb // Length}) // N
```

```
{0.0665802, 0.156529}
```

Data summaries, means etc.

Mean counts of site types. There is a marked asymmetry in the number of shared derived sites:

```
{meanpig = Mean[rawpig] // N, meanpig1kb = Mean[rawpig1kb] // N}
```

```
{{0.0453749, 456.442, 0.944318, 1.01752, 0.775523, 1.46288, 0.141291, 0.184794, 4.37266},
 {0.0862748, 904.445, 1.80625, 1.98222, 1.4354, 2.77362, 0.253676, 0.330954, 8.52645}}
```

93 and 84 % of blocks pass the 4-gamete test:

```
{len500 = pigfin1 // Flatten // Total, len1kb = pigfin11kb // Flatten // Total} /
  {rawpig11 // Length, rawpig111kb // Length} // N
```

```
{0.93342, 0.843471}
```

With 500b blocks, there are 4 mutations on average per block:

```
{{inmut = Total[Drop[Drop[meanpig, 2], -1]],
  outdis = Total[Drop[meanpig, 5]] / 2, meanlen = Total[Drop[meanpig, 1]]},
{inmut1kb = Total[Drop[Drop[meanpig1kb, 2], -1]],
  outdis1kb = Total[Drop[meanpig1kb, 5]] / 2, meanlen1kb = Total[Drop[meanpig1kb, 1]]}}
```

```
{{4.52633, 3.08082, 465.341}, {8.58213, 5.94235, 921.553}}
```

```
{meanlen * len500, meanlen1kb * len1kb}
```

$\{1.08133\times 10^{8},\ 1.75733\times 10^{8}\}$

About half of the loci are topologically informative. Loci that violate the 4-gamete criterion are automatically filtered out by the function configcount3stt. Less than 10% of loci fail the 4-gametes test (these are most likely back mutations).

```
{Table[pigfin1[[i]] // Flatten // Total, {i, 1, 4}] / (pigfin1 // Flatten // Total) // N,
 Table[pigfin11kb[[i]] // Flatten // Total, {i, 1, 4}] / (pigfin11kb // Flatten // Total) // N}
```

```
{{0.843037, 0.102168, 0.0108575, 0.043938}, {0.524883, 0.272507, 0.104766, 0.0978436}}
```

Instantaneous, unidirectional admixture (IUA)

Fitting a strict divergence model to the four datasets :

```
pigFIX1 = FindMaximum[{tripletLDurandtt[{θ, 1, 1}, {t1, t2}, {0, 0}, 3, pigfin1],
    {2 > θ > 1, 1 > t1 > 0 , 0.9 > t2 > 0 }}, {θ, t1, t2}]
```

$\{-1.1988\times 10^{6},\ \{\theta \to 1.49179,\ t1 \to 0.433056,\ t2 \to 0.737307\}\}$

```
pigFIX11kb = FindMaximum[{tripletLDurandtt[{θ, 1, 1}, {t1, t2}, {0, 0}, 3, pigfin11kb],
    {4 > θ > 2, 1 > t1 > 0 , 1.2 > t2 > 0.4}}, {θ, t1, t2}]
```

$\{-1.04965\times 10^{6},\ \{\theta \to 2.55069,\ t1 \to 0.553497,\ t2 \to 0.787398\}\}$

The IUA model fits better than the divergence model in all cases:

```
pigadm = FindMaximum[{tripletLDurandtt[{θ, 1, 1}, {t1, t2}, {tgf, f}, 3, pigfin1],
    { 2 > θ > 1, 0.5 > tgf > 0, 0.5 > f > 0, 1 > t1 > 0.2 , 1 > t2 > 0.2 }}, {θ, t1, t2, tgf, f}]
```

$\{-1.19436\times 10^{6},\ \{\theta \to 1.41944,\ t1 \to 0.252655,\ t2 \to 1.,\ tgf \to 0.241974,\ f \to 0.170247\}\}$

```
pigadm11kb = FindMaximum[{tripletLDurandtt[{θ, 1, 1}, {t1, t2}, {tgf, f}, 3, pigfin11kb],
   { 4 > θ > 2, 0.5 > tgf > 0, 0.5 > f > 0, 1 > t1 > 0.1 , 2 > t2 > 0.1}}, {θ, t1, t2, tgf, f}]
```

```
pigadmREV = FindMaximum[{tripletLDurandRevtt[{θ, 1, 1}, {t1, t2}, {tgf, f}, 3, pigfin1],
   { 2 > θ > 1, 0.5 > tgf > 0, 0.5 > f > 0, 1 > t1 > 0.1, 2 > t2 > 0.1 }}, {θ, t1, t2, tgf, f}]
```

$\{-1.19405\times 10^{6}$, {θ → 1.34818, t1 → 0.168608, t2 → 1.18535, tgf → 0.371811, f → 0.232657}}

```
pigadm11kbREV =
 FindMaximum[{tripletLDurandRevtt[{θ, 1, 1}, {t1, t2}, {tgf, f}, 3, pigfin11kb],
   {4 > θ > 2, 0.5 > tgf > 0, 0.5 > f > 0, 2 > t1 > 0.1, 2 > t2 > 0.1 }}, {θ, t1, t2, tgf, f}]
```

$\{-1.04181\times 10^{6}$, {θ → 2.28406, t1 → 0.250001, t2 → 1.20263, tgf → 0.425934, f → 0.169878}}

▫ *Instantaneous, bi-directional admixture (IBA)*

Symmetric admixture, i.e. $f_a = f_b$.

```
pigadmsym = FindMaximum[{tripletLDurandbothtt[{θ, f, f}, {t1, t2}, {tgf, empt}, 3, pigfin1],
   {2 > θ > 1, 0.8 > tgf > 0, 0.5 > f > 0, 0.6 > t1 > 0.01 , 2 > t2 > 0.6 }}, {θ, t1, t2, tgf, f}]
```

$\{-1.19488\times 10^{6}$, {θ → 1.26267, t1 → 0.0100006, t2 → 1.23963, tgf → 0.522149, f → 0.110787}}

```
pigadmsym1kb =
 FindMaximum[{tripletLDurandbothtt[{θ, f, f}, {t1, t2}, {tgf, empt}, 3, pigfin11kb],
   {3 > θ > 2, 1 > tgf > 0, 0.5 > f > 0, 1 > t1 > 0.2 , 2 > t2 > 0.6 }}, {θ, t1, t2, tgf, f}]
```

$\{-1.04257\times 10^{6}$, {θ → 2.18754, t1 → 0.2, t2 → 1.22502, tgf → 0.476282, f → 0.0766532}}

Asymmetric admixture:

```
pigadmboth =
 FindMaximum[{tripletLDurandbothtt[{θ, fa, fb}, {t1, t2}, {tgf, empt}, 3, pigfin1],
   {2 > θ > 1, 1 > tgf > 0.4, 0.4 > fa > 0, 0.4 > fb > 0, 0.4 > t1 > 0.01, 2 > t2 > 0.6}}, {θ,
   t1, t2, tgf, fa, fb}]
```

$\{-1.19388\times 10^{6}$,
{θ → 1.26328, t1 → 0.0701792, t2 → 1.3468, tgf → 0.505691, fa → 0.226632, fb → 0.0526494}}

```
pigadmboth1kb =
 FindMaximum[{tripletLDurandbothtt[{θ, fa, fb}, {t1, t2}, {tgf, empt}, 3, pigfin11kb],
   {3 > θ > 2, 1 > tgf > 0.4, 0.4 > fa > 0, 0.4 > fb > 0, 0.4 > t1 > 0.01, 2 > t2 > 0.6}}, {θ,
   t1, t2, tgf, fa, fb}]
```

$\{-1.04164\times 10^{6}$,
{θ → 2.19986, t1 → 0.190798, t2 → 1.28404, tgf → 0.50841, fa → 0.157408, fb → 0.0316529}}

▫ *Comparing models*

What is the difference in support between models? We are assuming that every 100th block is unlinked, so divide by 100...

```
modelpoint05kb = {pigFIX1, pigadm, pigadmREV, pigadmsym, pigadmboth};
modelComp05kb = {{"Div", "IUA(S→v)", "IUA(V->S)", "ISA", "IBA"},
  SetPrecision[Chop[(pigadmboth⟦1⟧ - (#⟦1⟧ & /@modelpoint05kb)) / 100, 10^-6], 3]};
modelComp05kb // TableForm
```

Div	IUA(S→v)	IUA(V->S)	ISA	IBA
49.3	22.8	1.77	10.1	0

```
modelpoint1kb = {pigFIX11kb, pigadm11kb, pigadm11kbREV, pigadmsym1kb, pigadmboth1kb};
modelComp1kb = {{"Div", "IUA(S→v)", "IUA(V->S)", "ISA", "IBA"},
  SetPrecision[Chop[(pigadmboth1kb[[1]] - (#[[1]] & /@modelpoint1kb)) / 100, 10^-6], 3]};
modelComp1kb // TableForm
```

Div	IUA(S→v)	IUA(V->S)	ISA	IBA
80.1	26.8	1.68	9.24	0

Saving the MLE solutions for each model:

```
Export["/home/konrad/Manuscripts/PigPhylogeography/Pig_data/modelComp05kb06_05.txt",
  modelComp05kb];
Export["/home/konrad/Manuscripts/PigPhylogeography/Pig_data/modelComp1kb06_05.txt",
  modelComp1kb];

Export["/home/konrad/Manuscripts/PigPhylogeography/Pig_data/modelpoint05kb06_05.txt",
  modelpoint05kb];
Export["/home/konrad/Manuscripts/PigPhylogeography/Pig_data/modelpoint1kb06_05.txt",
  modelpoint1kb];

modelComp05kbt = ReadList[
   "/home/konrad/Manuscripts/PigPhylogeography/Pig_data/modelComp05kb06_05.txt"];
modelComp1kbt = ReadList[
   "/home/konrad/Manuscripts/PigPhylogeography/Pig_data/modelComp1kb06_05.txt"];

modelpoint05kb = ReadList[
   "/home/konrad/Manuscripts/PigPhylogeography/Pig_data/modelpoint05kb06_05.txt"];
modelpoint1kb = ReadList[
   "/home/konrad/Manuscripts/PigPhylogeography/Pig_data/modelpoint1kb06_05.txt"];
```

Marginal support for individual parameters

admref, 0.5kb

Assuming a generation time of 5 years and a divergence from the outgroup 10.5 MYA,the (per generation per block) mutation rate is:

```
{outμ = outdis / (10 500 000 / 5), outμ1kb = outdis1kb / (10 500 000 / 5)}
```

$\{1.46706 \times 10^{-6}, 2.82969 \times 10^{-6}\}$

```
scaltgf = Table[{t, (2 t outμ ) / 5}, {t, 0, 1 500 000, 100 000}];
scalT1 = Table[{t, (2 t outμ ) / 5}, {t, 600 000, 1 600 000, 50 000}];
scalT2 = Table[{t, (2 t outμ) / 5}, {t, 2 200 000, 4 500 000, 50 000}];

pigadmREV = FindMaximum[{tripletLDurandRevtt[{θ, 1, 1}, {t1, t2}, {tgf, f}, 3, pigfin1],
    { 2 > θ > 1, 0.5 > tgf > 0, 0.5 > f > 0, 1 > t1 > 0.1, 2 > t2 > 0.1 }}, {θ, t1, t2, tgf, f}]
```

$\{-1.19405 \times 10^{6}, \{\theta \to 1.34818, t1 \to 0.168608, t2 \to 1.18535, tgf \to 0.371811, f \to 0.232657\}\}$

```
ftabadmRev = ParallelTable[
  {f, FindMaximum[{tripletLDurandRevtt[{θ, 1, 1}, {t1, t2}, {tgf, f}, 3, pigfin1],
      {1.8 > θ > 1, 0.6 > tgf > 0.1, 0.4 > t1 > 0.01, 1.6 > t2 > 0.6}},
     {θ, t1, t2, tgf}]}, {f, 0.15, 0.3, 0.01}]

tgftabadmRev =
  ParallelTable[{scaltgf[[i, 1]], FindMaximum[{tripletLDurandRevtt[{θ, 1, 1}, {t1, t2},
       {scaltgf[[i, 2]] / θ, f}, 3, pigfin1], {1.8 > θ > 1, 0.35 > f > 0, 0.4 > t1 > 0.01,
       1.6 > t2 > 0.6}}, {θ, t1, t2, f}]}, {i, 2, scaltgf // Length}];
```

```
T1tabadmRev = ParallelTable[{scalT1⟦i, 1⟧,
    FindMaximum[{tripletLDurandRevtt[{θ, 1, 1}, {((scalT1 ⟦i, 2⟧) / θ) - tgf, t2},
       {tgf, f}, 3, pigfin1], {1.8 > θ > 1, 0.35 > f > 0, 0.6 > tgf > 0.1, 1.6 > t2 > 0.6}},
     {θ, tgf, t2, f}]}, {i, 1, scalT1 // Length}];
```

```
T2tabadmRev = ParallelTable[{scalT2 ⟦i, 1⟧,
    FindMaximum[{tripletLDurandRevtt[{θ, 1, 1}, {t1, ((scalT2 ⟦i, 2⟧) / θ) - (tgf + t1)},
       {tgf, f}, 3, pigfin1], {1.8 > θ > 1, 0.35 > f > 0, 0.6 > tgf > 0.1, 0.4 > t1 > 0.01}},
     {θ, tgf, t1, f}]}, {i, 1, scalT2 // Length}];
```

```
marglnL05Rev = {ftabadmRev, tgftabadmRev, T1tabadmRev, T2tabadmRev};
Export["/home/konrad/Manuscripts/PigPhylogeography/Pig_data/marglnL05Rev15_05.txt",
  marglnL05Rev];
```

```
marglnL05Rev =
  ReadList["/home/konrad/Manuscripts/PigPhylogeography/Pig_data/marglnL05Rev15_05.txt"];
```

- ***admboth, 0.5kb***
- ***admboth, 1kb***
- ***Plots***

```
<< "Graphics`Graphics`"(*to get UnitScale*);
conff = {{0, -3.841 / 2}, {5, -3.841 / 2}};
xxticksf = (Append[#1, {0, 0.02}] &) /@UnitScale[0.05, 0.3, 0.05];
yyticksf = (Append[#1, {0, 0.02}] &) /@UnitScale[-7, -1, 1];
kllegendf = {Graphics[{Thick, Red, Line[{{-2, 0.5}, {2, 0.5}}]}],
   Graphics[{Thick, Blue, Line[{{-2, 0.5}, {2, 0.5}}]}]};
```

```
fplot = ListPlot[{conff, {#⟦1⟧, (#⟦2, 1⟧ - pigadmboth⟦1⟧) / 100} & /@marglnL05kb[[1, 1]],
    {#⟦1⟧, (#⟦2, 1⟧ - pigadmboth⟦1⟧) / 100} & /@marglnL05kb[[1, 2]],
    {#⟦1⟧, (#⟦2, 1⟧ - pigadmREV⟦1⟧) / 100} & /@marglnL05Rev[[1]]},
  Joined → True, Frame → {False, True, True, False}, Axes → False,
  PlotRange -> {{0, 0.3}, {0, -7}}, FrameTicks → {{}, yyticksf, xxticksf, {}},
  Epilog → {makePlotLegend[{"fV→S", "fS→V"}, kllegendf, {0.9, 0.3}, 14, 12, "Arial"],
    Text["ΔlnL", {0, 0.2}], Text["f", {0.31, 0}]},
  PlotStyle -> {{Dashed}, {Red, Thick}, {Blue, Thick}, {Red, Thick, Dashed}},
  AxesLabel -> {"f", "ΔlnL"}, PlotRangeClipping → False,
  ImagePadding → {{20, 20}, {20, 20}}]
```

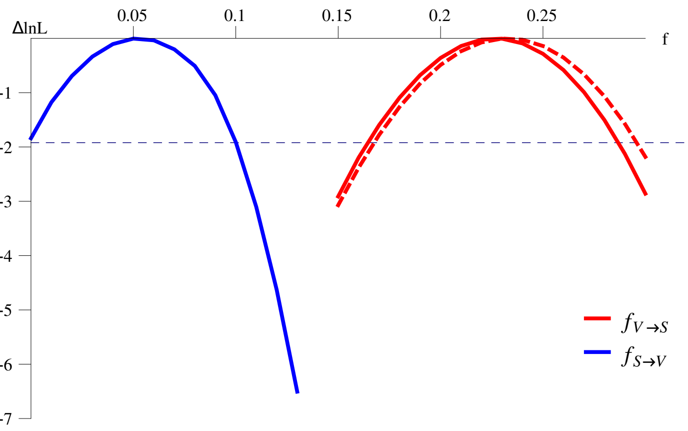


```
Export["/home/konrad/Manuscripts/PigPhylogeography/Pig_figs/fvslnL_plot.pdf", fplot];
```

```
fplot1kb = ListPlot[{conff, {#[[1]], (#[[2, 1]] - pigadmboth[[1]]) / 100} & /@marglnL05kb[[1, 1]],
    {#[[1]], (#[[2, 1]] - pigadmboth[[1]]) / 100} & /@marglnL05kb[[1, 2]],
    {#[[1]], (#[[2, 1]] - pigadmboth1kb[[1]]) / 100} & /@marglnL1kb[[1]]},
  Joined → True, Frame → {False, True, True, False}, Axes → False,
  PlotRange -> {{0, 0.3}, {0, -7}}, FrameTicks → {{}, yyticksf, xxticksf, {}},
  Epilog → {makePlotLegend[{"fV→S", "fS→V"}, kllegendf, {0.9, 0.3}, 14, 12, "Arial"],
    Text["ΔlnL", {0, 0.2}], Text["f", {0.31, 0}]},
  PlotStyle -> {{Dashed}, {Red, Thick}, {Blue, Thick}, {Red, Thick, Dashed}},
  AxesLabel -> {"f", "ΔlnL"}, PlotRangeClipping → False,
  ImagePadding → {{20, 20}, {20, 20}}]
```

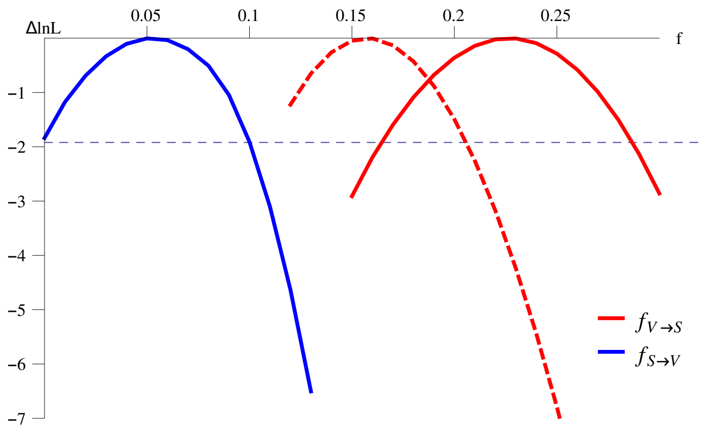


```
Export[
  "/home/konrad/Manuscripts/PigPhylogeography/Pig_figs/fvslnL_plot1kb.pdf", fplot1kb];
```

```
xxticks = (Append[#1, {0, 0.02}] &) /@UnitScale[0, 6, 1];
yyticks = (Append[#1, {0, 0.02}] &) /@UnitScale[-10, -1, 1];
kllegend = {Graphics[{Thick, Red, Line[{{-2, 0.5}, {2, 0.5}}]}],
    Graphics[{Thick, Blue, Line[{{-2, 0.5}, {2, 0.5}}]}],
    Graphics[{Thick, Black, Line[{{-2, 0}, {2, 0}}]}]};
```

```
timeplot = ListPlot[
  {conff, {#[[1]] / 10^6, (#[[2, 1]] - pigadmboth[[1]]) / 100} & /@ marglnL05kb[[1, 3]],
   {#[[1]] / 10^6, (#[[2, 1]] - pigadmboth[[1]]) / 100} & /@ marglnL05kb[[1, 4]],
   {#[[1]] / 10^6, (#[[2, 1]] - pigadmboth[[1]]) / 100} & /@ marglnL05kb[[1, 5]],
   {#⟦1⟧ / 10^6, (#⟦2, 1⟧ - pigadmREV⟦1⟧) / 100} & /@marglnL05Rev[[2]],
   {#⟦1⟧ / 10^6, (#⟦2, 1⟧ - pigadmREV⟦1⟧) / 100} & /@marglnL05Rev[[3]],
   {#⟦1⟧ / 10^6, (#⟦2, 1⟧ - pigadmREV⟦1⟧) / 100} & /@marglnL05Rev[[4]]},
  Joined -> True, Frame → {False, True, True, False}, Axes → False,
  PlotRange -> {{0, 4.6}, {0, -10}}, FrameTicks → {{}, yyticks, xxticks, {}},
  Epilog → {makePlotLegend[{"t_gf", "T_1", "T_2"}, kllegend, {0.9, 0.3}, 14, 12, "Arial"],
    Text["MY", {4.75, 0}], Text["ΔlnL", {-0, 1.4}]},
  PlotStyle -> {{Dashed}, {Red, Thick}, {Blue, Thick}, {Black, Thick},
    {Red, Dashed, Thick}, {Blue, Dashed, Thick}, {Black, Dashed, Thick}},
  AxesLabel -> {"MY", "ΔlnL"}, PlotRangeClipping → False,
  ImagePadding → {{20, 20}, {20, 20}}]
```

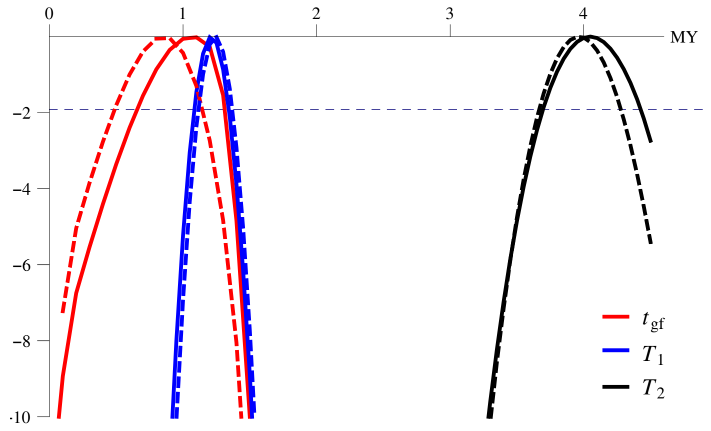


```
Export[
  "/home/konrad/Manuscripts/PigPhylogeography/Pig_figs/timesvslnL_plot.pdf", timeplot];
```

■ Model fit

▫ *External mutations conditional on topology*

▫ *Expected D*

What is E[D] under the three different models?

```
expD = ((expBaba - expAbba) / (expAbba + expBaba));
{expD /. (pigadmboth[[2]] /. t1 → T[{1}] /. t2 → T[{2}]),
 expD /. (pigadm[[2]] /. f → fb /. t1 → T[{1}] /. t2 → T[{2}]) /. fa → 0,
 expD /. (pigadmREV[[2]] /. f → fa /. t1 → T[{1}] /. t2 → T[{2}]) /. fb → 0}
```

```
{0.158386, 0.215037, 0.120102}
```

We restrict the D stats computation to blocks that pass the 4-gamete test:

```
fourgam05 = Select[rawpig11, #⟦5⟧ > 0 && #⟦3⟧ == 0 && #⟦6⟧ == 0 ||
     #⟦3⟧ > 0 && #⟦5⟧ == 0 && #⟦6⟧ == 0 || #⟦6⟧ > 0 && #⟦3⟧ == 0 && #⟦5⟧ == 0 &];
fourgam1 = Select[rawpig111kb, #⟦5⟧ > 0 && #⟦3⟧ == 0 && #⟦6⟧ == 0 ||
     #⟦3⟧ > 0 && #⟦5⟧ == 0 && #⟦6⟧ == 0 || #⟦6⟧ > 0 && #⟦3⟧ == 0 && #⟦5⟧ == 0 &];
```

D is lower for all the block data (raw) than the 4-gamete filtered blocks:

```
{{" ", "raw", "4-gamete"}, {"0.5kb", ((rawpig11 // Total)[[5]] - (rawpig11 // Total)[[3]]) /
     ((rawpig11 // Total)[[5]] + (rawpig11 // Total)[[3]]) // N,
   ((fourgam05 // Total)[[5]] - (fourgam05 // Total)[[3]]) /
     ((fourgam05 // Total)[[5]] + (fourgam05 // Total)[[3]]) // N},
  {"1kb", ((rawpig111kb // Total)[[5]] - (rawpig111kb // Total)[[3]]) /
     ((rawpig111kb // Total)[[5]] + (rawpig111kb // Total)[[3]]) // N,
   ((fourgam1 // Total)[[5]] - (fourgam1 // Total)[[3]]) /
     ((fourgam1 // Total)[[5]] + (fourgam1 // Total)[[3]]) // N}} // TableForm
```

	raw	4-gamete
0.5kb	0.133411	0.175552
1kb	0.132183	0.215107

The estimated admixture fraction actually goes down in the 1 kb data despite the fact that D goes up. Counter-intuitively, E[D] goes down with increasing f_a. E[D] for 0.5kb (solid) and 1 kb (dashed) data under the model of bidirectional admixture.

```
expAbba =
  ((-D[neanderDurandbothGFacαβ /. ω[{_}] → 0, ω[{a, c}]]) /. ω[{a, c}] → 0) // Simplify;
expBaba = ((-D[neanderDurandbothGFabαβ /. ω[{_}] → 0, ω[{a, b}]]) /. ω[{a, b}] → 0) //
   Simplify;
```

```
Plot[{((expBaba - expAbba) / (expAbba + expBaba)) /. T[{1}] → pigadmboth1kb[[2, 2, 2]] /.
    T[{2}] → pigadmboth1kb[[2, 3, 2]] /. fb → pigadmboth1kb[[2, -1, 2]],
  ((expBaba - expAbba) / (expAbba + expBaba)) /. T[{1}] → pigadmboth[[2, 2, 2]] /.
    T[{2}] → pigadmboth[[2, 3, 2]] /. fb → pigadmboth[[2, -1, 2]]},
 {fa, 0.1, 0.4}, PlotStyle → {{Dashed, Thick}, Thick}, AxesLabel → {"f", "E[D]"}]
```

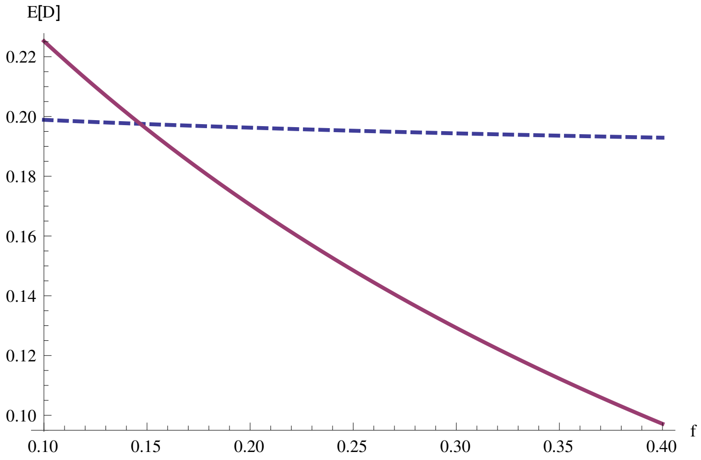

Generating functions and definitions

- **GF definitions**
- **lik2dFull**
- **lik2dMarg**
- **lik1dMarg**
- **likMargTot, likNoTopTot**
- **tripletLDurandbothtt, tripletLDurandtt, tripletLDurandttREV**
- **pr3s**
- **sitecount3s**

- **configcount3stt**
- **exhist**
- **corret**
- **makePlotLegend**
